# From daily burden to scheduled protection: the “vaccine-like” shift in hypertension

**DOI:** 10.1038/s41371-026-01149-2

**Published:** 2026-04-25

**Authors:** Lucas Maciel de Almeida Corrêa, Gabriel Rian Mazur, Clara Belo Gamon Santiago, Alexandre de Assis Barbosa, Luiggi Kevin Virgino Brandão, Gabriel Costa de Santana, Letícia Esteves Dante

**Affiliations:** 1 Faculdade de Medicina de São José do Rio Preto (FAMERP), São José do Rio Preto, Brazil; 2 Pontifícia Universidade Católica do Paraná (PUCPR), Curitiba, Brazil; 3 Universidade Estadual de Mato Grosso do Sul (UEMS), Campo Grande, Brazil; 4 Centro Universitário Uninorte (UNINORTE), Rio Branco, Brazil; 5 Universidade Salvador (UNIFACS), Salvador, Brazil

**Keywords:** Preventive medicine, Hypertension

## Abstract

- Global hypertension control remains stagnant due to the fragility of daily medication adherence rather than a lack of effective drugs.
- Long-acting siRNA therapies offer a “vaccine-like” paradigm shift, transferring responsibility from the patient’s daily memory to the health system’s reliability.
- This approach introduces “pharmacological moral hazard,” where patients may neglect lifestyle modifications under a false sense of total security.
- Realizing the promise of democratized protection requires redesigning care pathways to ensure that infrequent dosing does not lead to clinical disengagement.

## The architecture of failure: why daily adherence breaks down

Hypertension is one of medicine’s most solvable problems and one of global health’s most persistent failures [1–3]. Effective, inexpensive therapies exist, yet control rates remain low: in pooled global analyses of 1990-2019 trends, fewer than one-quarter of people with hypertension had controlled blood pressure (BP) in 2019 [1, 3]. At the same time, elevated systolic BP remains the leading modifiable risk factor for death and disability worldwide [2]. This disconnect implies that the limiting step is often not pharmacology, but the architecture of long-term care itself [3–5].

The core vulnerability is that BP control has been designed as a daily behavioral achievement [4–6]. Because hypertension is frequently asymptomatic, there is little immediate feedback to reinforce pill-taking, and adherence erodes across initiation, implementation, and persistence [4, 6]. Even when access is secure, real-world routines are fragile; interruptions accumulate as treatment competes with work, caregiving, travel, and polypharmacy, and adherence can deteriorate over time [4, 5, 7]. Psychosocial comorbidity, including depression, further widens the gap between efficacy and effectiveness [8].

This is why the concepts of treatment burden and “care work” matter [9, 10]. Chronic disease management is not only taking tablets; it is scheduling, monitoring, refilling, attending visits, and continuously organizing life around prevention [9, 10]. When health systems presume stable time, attention, transportation, and continuity, they effectively outsource therapeutic success to a patient’s capacity to sustain thousands of micro-decisions [9, 10]. In that model, adherence becomes an implicit social filter: cardiovascular protection tracks life stability as much as clinical need, intersecting with the social determinants of health and broader inequities [11, 12].

## From pills to protection: designing adherence out of BP control

Long-acting RNA interference (RNAi) introduces a different design principle: instead of asking patients to remember therapy, make therapy difficult to forget [13–16]. Zilebesiran, a small interfering RNA (siRNA) targeting hepatic angiotensinogen, suppresses the upstream substrate of the renin-angiotensin-aldosterone system (RAAS), enabling sustained BP lowering for months after a single subcutaneous dose [13]. In KARDIA-1, dosing every 3 months or every 6 months produced durable reductions in systolic BP, aligning pharmacologic coverage with a cadence that is intrinsically less dependent on day-to-day behavior [14]. This pharmacology has catalyzed the “vaccine-like” proposal: BP control as scheduled protection rather than continuous pill-taking [15, 16].

**Fig. 1:**
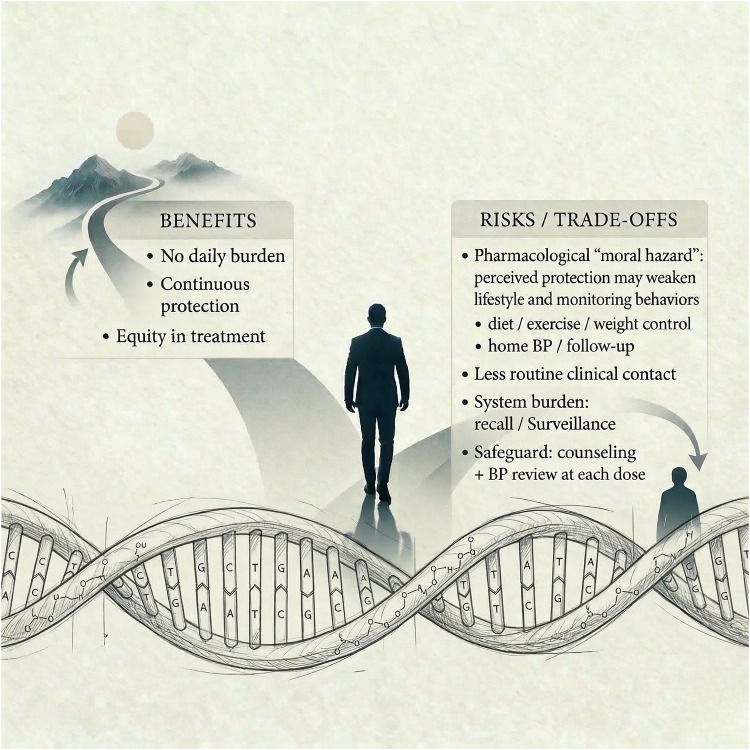
The therapeutic trade-off: while RNAi removes the daily burden of adherence, it may introduce behavioral risks that require redesign of care. Original figure by the authors.

The most consequential shift is not molecular; it is operational [15, 16]. In a vaccine-like model, adherence becomes appointment-based and system-mediated [15–17]. A more appropriate clinical analogue is scheduled administration of infusion-based biologic therapies, where effectiveness is less dependent on daily patient action and more dependent on reliable return for repeated administration, monitoring, and continuity of care [17]. Similarly, siRNA antihypertensives could reduce the variance in BP control created by missed doses, fragmented routines, and fatigue with chronic regimens, potentially narrowing the gap between trial efficacy and population effectiveness [5, 14–16]. This therapeutic trade-off is summarized in Fig. 1 [15, 16].

But long-acting therapy also relocates risk [17–20]. When dosing is infrequent, the patient’s day-to-day task load decreases, yet the health system must reliably perform recall, outreach, documentation, and longitudinal safety surveillance [17–20]. Across chronic diseases, adherence support interventions are often modest or inconsistent, highlighting that durable control depends on system design as much as patient intention [18, 19]. Implementation lessons from long-acting antiviral strategies underscore similar challenges: maintaining follow-up, preventing missed doses from becoming “silent lapses,” and ensuring services can deliver continuity at scale [20].

The second trade-off is behavioral: if patients perceive months-long pharmacologic protection, engagement with lifestyle measures and routine self-management may weaken, although this remains a plausible concern rather than a directly demonstrated effect in long-acting siRNA antihypertensive therapy [21–23]. In this context, “moral hazard” refers to the tendency for perceived protection to reduce engagement in other preventive behaviors [21]. For hypertension specifically, one plausible form of “pharmacological moral hazard” would be reduced emphasis on sodium restriction, weight management, physical activity, and home BP monitoring, which could weaken an important feedback loop for self-management and make routine ascertainment of true control more difficult [21–23]. The broader concern is familiar: perceived protection can encourage overreliance on technology and underinvestment in complementary preventive behaviors [21]. Infrequent injections may also reduce the number of routine contact moments that often serve as opportunities for education, shared decision-making, and risk-factor review, unless these touchpoints are deliberately redesigned [17–19].

These vulnerabilities are not arguments against siRNA; they are design specifications for care pathways [18, 19, 21, 23]. In hypertension, durable therapy should not come at the price of making BP itself less visible to patients or clinicians [23]. If the injection becomes a twice-yearly ritual, it should be engineered as a high-value touchpoint: structured lifestyle reinforcement, review of home BP data, medication reconciliation, and proactive monitoring for adverse events [19, 23]. In other words, the goal is not to replace a daily reminder with silence, but to replace fragile daily adherence with a dependable clinical scaffold [18, 19].

## Beyond the molecule: redefining prevention as reliability

The vaccine-like strategy for hypertension is best understood as a reallocation of responsibility: from the patient’s memory to the health system’s reliability [15, 16]. By decoupling BP control from daily execution, siRNA therapeutics could democratize cardiovascular protection and blunt the adherence trap that has long undermined population-level control [1, 5, 13].

Yet the long-acting promise will be realized only if we resist “fire-and-forget” thinking [21]. RAAS silencing can stabilize hemodynamics, but it does not automatically cultivate healthier diets, activity patterns, or sustained engagement with preventive care [13, 21, 22]. The decisive question is therefore structural, not molecular: will we treat the injection as a cure that ends dialogue, or as a security floor that creates room for better dialogue? [21]

If care systems can build dependable recall, surveillance, and human-centered follow-up around infrequent dosing, the vaccine-like model may do more than lower BP; it may test whether prevention can be made more durable without making hypertension less visible to patients or clinicians [18, 19]. This reframing is now entering prospective clinical testing [24]. Importantly, the available phase 2 evidence in higher-risk patients remains mixed rather than definitive. In KARDIA-3, placebo-adjusted office systolic BP lowering at the month-3 primary endpoint did not meet the prespecified definition for statistical significance after multiplicity adjustment, although the study still helped define the dose, target population, and phase 3 design carried forward into ZENITH [24, 25]. As zilebesiran moves into ZENITH, a global phase 3, event-driven trial expected to enroll approximately 11,000 patients, the field is moving beyond BP durability alone toward the harder question of whether twice-yearly angiotensinogen silencing can reduce cardiovascular death, nonfatal myocardial infarction, nonfatal stroke, or heart failure events when added to standard care [24]. Whether such long-acting treatment might also inadvertently reduce engagement with BP monitoring and broader risk management therefore remains a clinically relevant concern, even if it is not itself a direct trial endpoint.
